# Locational effects on oral microbiota among long-term care patients

**DOI:** 10.1080/20002297.2022.2033003

**Published:** 2022-02-14

**Authors:** Fa-Tzu Tsai, Ding-Han Wang, Cheng-Chieh Yang, Yu-Cheng Lin, Lin-Jack Huang, Wei-Yu Tsai, Chang-Wei Li, Wun-Eng Hsu, Hsi-Feng Tu, Ming-Lun Hsu

**Affiliations:** a Institute of Oral Biology, National Yang Ming Chiao Tung University, Taipei, Taiwan; b Department of Dentistry, National Yang Ming Chiao Tung University, Taipei, Taiwan; c Department of Stomatology, Oral & Maxillofacial Surgery, Taipei Veterans General Hospital, Taipei, Taiwan; d Department of Dentistry, National Yang Ming Chiao Tung University Hospital, Yilan, Taiwan; e Department of Biological Science and Technology, National Yang Ming Chiao Tung University, Hsinchu, Taiwan; f Department of Dentistry, Far Eastern Memorial Hospital, New Taipei City, Taiwan

**Keywords:** Oral microbiota, next-generation sequencing (NGS), long-term care patients, home care, outpatient department (OPD)

## Abstract

**Background:**

Dysbiosis of oral microbiota is the cause of many diseases related to oral and general health. However, few Asia-based studies have evaluated the role of oral microbiota in patients receiving long-term care. Thus, new indications are needed for early prevention and risk management based on information derived from the oral microbiota.

**Methods:**

We used next-generation sequencing (NGS) to identify the oral bacterial composition and abundance in patients receiving long-term care: 20 from the outpatient department (OPD) and 20 home-care patients. Their microbial compositions, taxonomy, and alpha/beta diversity were characterized.

**Results:**

Microbiota from the two groups showed different diversity and homogeneity, as well as distinct bacterial species. A more diverse and stable microbial population was observed among OPD patients. Our findings indicated that home-care patients had a higher risk of oral diseases due to the existence of dominant species and a less stable microbial community.

**Conclusion:**

This work was the first in Taiwan to use NGS to investigate the oral microbiota of long-term care patients. Our study demonstrated the potential use of dominant bacterial species as biomarkers for the risk management of posttreatment complications.

## Introduction

In the past, a single strain of bacteria was often viewed as the sole pathogen of an infectious disease [1]; however, in many cases, the composition of the microbiota is the primary disease-causing factor [2,3]. Several studies have indicated that an imbalanced gut microbiota was the primary factor for type 2 diabetes, obesity, and cancer [2–7]. Concordantly, dysbiosis of the microbiota was found to cause several oral and gastrointestinal diseases, including periodontal disease, inflammatory bowel diseases (IBDs) and irritable bowel syndrome (IBS) [7–10]. The oral microbiota is the second biggest microbiota in the human body [11]. Consequently, composition of oral microbiota may influence not only oral health, but also general health conditions [12]. The importance of a healthy oral microbiota is particularly relevant in some community-based elderly populations, especially for long-term care patients who suffer from frailty and chronic diseases [13–15].

Treatment of chronic diseases has been transformed by the development of precision medicine in recent years [16]. On the other hand, precision medicine also promoted the efficacy of disease prevention through the detection of specific host or microbial genes [17,18]. Disease risks could thus be evaluated by comparing healthy versus diseased individuals [16,19]. The advancement of precision medicine was mainly driven by next generation sequencing [20,21], which has been used to investigate the microbiota for diagnosis of infectious diseases and risk assessment.

Geriatric patients are inherently more susceptible to infectious diseases. For example, studies have shown that these patients, especially those who are bedridden, have a higher risk of pneumonia [22,23], even despite the provision of oral health care [24,25]. For debilitated patients, feeding methods can dramatically modify the oral microbiome [26,27]. Given the variation and roles of oral microbiome, identical antibiotic treatment regimens could result in vastly different outcomes at different localities, e.g. nursing home, residential, and hospital patients [28,29].

As antibiotic treatments of long-term care patients were influenced by different localities (e.g. nursing home vs. hospital), it is possible that lifestyle factors drive differences in the oral microbiota and play a crucial role in infection. In this regard, one particular lifestyle-affected population is home-care patients, whose oral hygiene maintenance is extremely challenging [22–24]. In contrast, outpatients (i.e. the ‘control’ group) are usually less susceptible to hygiene-related problems. It is therefore conceivable that modifying dental treatments based on these findings on oral microbiota may minimize risks (e.g. pneumonia) and improve life quality and safety of long-term patients who receive home care. However, few studies have evaluated this locality issue in the Asia-Pacific region, especially in Taiwan.

In this study, we hypothesized that the composition of oral microbiota between OPD and home-care patients was different and may play a decisive role in oral health. We leveraged the power of next-generation sequencing to identify the bacterial composition and abundance in the oral cavity, and characterized the oral microbiota of these long-term care patients. We found that oral microbiota of home-care patients presented significantly less species diversity, and lower homogeneity of species abundance, compared to outpatients. Our findings indicated that dysbiosis of oral microbiota in disabled patients could potentially cause the unhealthy status of general and oral conditions. Identifying properties of oral microbiota among different patient groups may inform new criteria and indications for early prevention and risk management.

## Materials and methods

### Study subjects, study design, and sample collection

We included 40 patients receiving long-term care: 20 from the dental outpatient department (OPD) related to special needs of the hospital of National Yang Ming Chao Tung University and 20 community-dwelling patients who had registered for the dental home-care service in Yilan County. Home-care patients were those who had disabilities and needed long-term care, but they could not visit the hospital for treatments. Hospital patients were those who also had disabilities but could visit the hospital by themselves. Before sample collection, patients were requested to fill out a questionnaire on basic clinical-demographic information, including age, sex, systemic diseases, nasogastric tube indwelling, history of pneumonia, and eating habits. Informed consent was obtained from all patients. The oral characteristic of the patients was recorded by the dentist before the tongue swab. Next, a cotton swab was used to collect their tongue plaque samples on the surface of the tongue, and NGS was used to analyze the microbiota composition in each sample. This study was approved by the Ethics Committee of the hospital of National Yang Ming Chiao Tung University, Taiwan (2020B002).

### Oral microbiota profiling with 16S rDNA sequencing

After the tongue plaque collecting, the samples were stored at 4°C in the ice bucket while transported to the laboratory and extracted the sample into genome DNA in 24 hours. Total genome DNA from samples was extracted according to manufacturer’s protocols. DNA concentration was monitored by Equalbit dsDNA HS Assay Kit, and the samples were stored at −20°C until further analysis. The V3 and V4 hypervariable regions of prokaryotic 16S rDNA were selected for generating amplicons and following taxonomy analysis. The panel of proprietary primers aimed at relatively conserved regions bordering the V3 and V4 hypervariable regions of bacteria and Archaea16S rDNA was used. Forward and reverse primers were 341 F (CCTACGGGNGGCWGCAG) and 805 R (GACTACHVGGGTATCTAATCC), respectively. Then, a linker with Index is added to the end of the PCR product of 16S rDNA by PCR for NGS sequencing. The library was purified with magnetic beads, and the concentration was detected by a microplate reader and the fragment size was detected by agarose gel electrophoresis. The library was quantified to 10 nM, and 150PE (paired-end) sequencing was performed according to the Illumina MiSeq (Illumina, San Diego, CA, USA) instrument manual.

### Bioinformatics analysis

Sequencing reads from different samples were identified and separated by the Index sequence. Paired-end reads were joined together, and primer and adapter sequences were removed by cutadapt (v1.9.1). The 5’ and 3’ bases with Q score lower than 20 were also removed for each sequence, and the sequences with length larger than 200 bp were retained. Chimeric sequences were then removed to obtain the effective sequences for cluster analysis using VSEARCH (v1.9.6), and the resulting representative sequences was applied to OTU (Operational Taxonomic Unit) clustering using QIIME (v1.9.1). A 97% similarity standard was applied to V3-V4 sequence clusters. QIIME and the SILVA ribosomal RNA sequence database (release 132) were used for taxonomy assignment of OTU representative sequences. After taxonomy assignment, random sampling was applied to flatten the number of sequences of all samples. The OTU table of raw counts was normalized to an OTU table of relative abundance values. Taxa of the same type were agglomerated at the phylum, class, order, family, genus and species levels via the SILVA database.

Biodiversity was compared between classified groups using the nonparametric Wilcoxon test. Shannon index, Chao1 index, abundance-based coverage estimators (ACE), Simpson index and Rarefaction curves were calculated to investigate the species evenness and richness. Phylogeny-based UniFrac analysis was also performed to realize the diversity and the degrees of differences among samples using QIIME. Clustering analyses, such as principal components analysis (PCA), principal coordinates analysis (PCoA) and non-metric multidimensional scaling (NMDS), were used for visualization based on data reduction of patterns in an n-dimensional dataset. PCA is based on the OTU abundances of samples; PCoA and NMDS are based on the Bray-Curtis distance matrix between samples. Anosim analysis compared the differences between the analysis group rank values and rank within the group differences between whether group has significance. LEfSe (Linear discriminant analysis Effect Size) analysis compared the differences between the species in different groups. Analysis and graph plotting of biodiversity, clustering analyses (PCA, PCoA, NMDS), Anosim analysis were processed in the R software (v3.3.1, R foundation, Vienna, Austria). LEfSe analysis was processed in LEfSe 1.0.

## Results

### Patient characteristics and oral examination results

**Figure 1.**
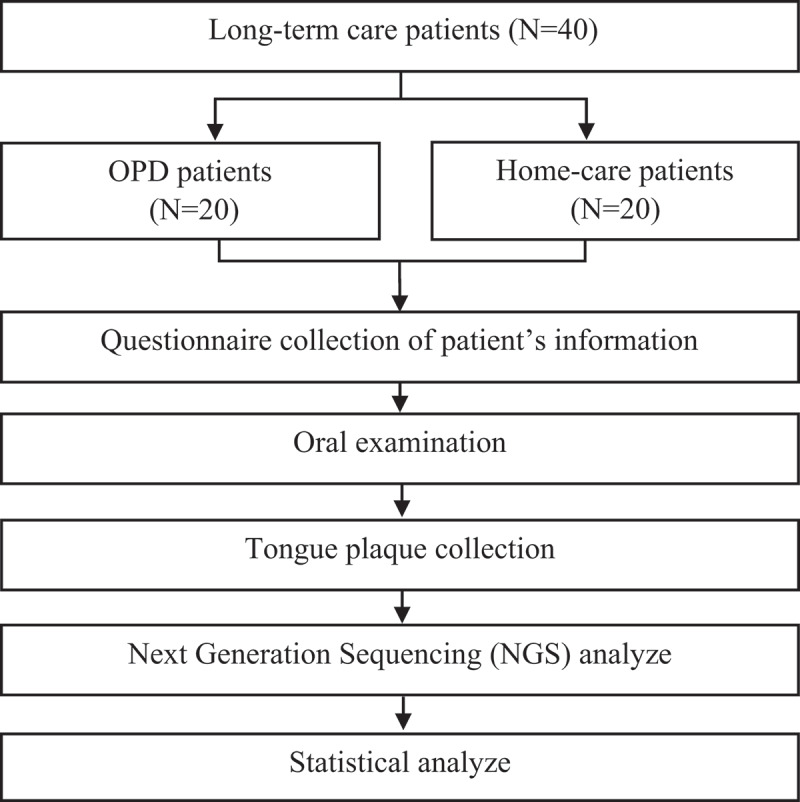
Flowchart of the study.

The study flowchart is presented in Figure 1; patient characteristics and the result of oral examination are summarized in Table 1. Patients presented different disability levels, ranging from moderate to profound, and more than one type of systemic disease. In general, most of the patients from home-care had worse oral hygiene and periodontal health condition, e.g. more calculus and residual roots, compared to the OPD patients (Table 1).

**Table 1.** Demographic characteristics and oral examination results of OPD (n = 20) and homecare (n = 20) patients

		OPD	Home-care
Number of patients		20	20
Male/Female		10/10	8 /12
Age in years mean (SD)		61.15 (15)	71.65 (25.7)
Disability level			
	Moderate	7	0
	Severe	8	10
	Profound	5	10
Diseases			
	Hypertension	8	10
	Diabetes mellitus	6	5
	Cardiovascular disease	5	10
	Dementia	2	9
	Liver disease	3	3
	Kidney disease	3	6
Pneumonia history			
	Yes	2	11
	No	18	9
Nasogastric tube			
	Yes	3	13
	No	17	7
Bedridden			
	Yes	2	19
	No	18	1
Caries experience			
	Decay	6	2
	Missing	3	7
Residual root			
	Yes	5	11
	No	15	9
Periodontal status			
	Healthy	4	0
	Gingivitis	8	0
	Periodontitis	8	20
Calculus			
	Mild	2	0
	Moderate	0	1
	Severe	5	16
Moveable denture			
	Partial	2	0
	Full mouth	3	0
Crown/Bridge		3	10

**Figure 2.**
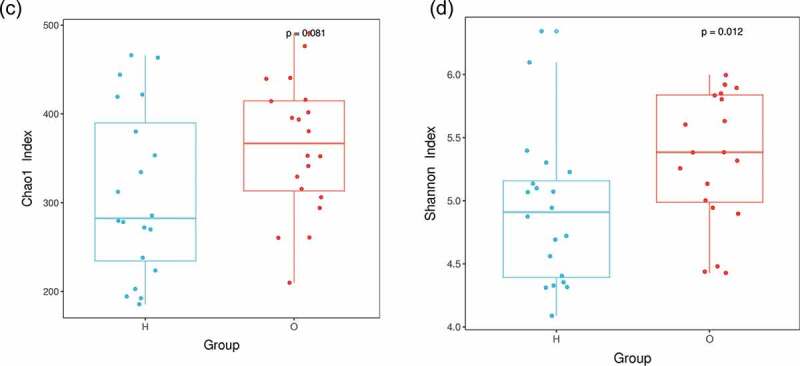
Comparison of the microbiota composition between OPD and home-care groups. (a) Venn diagram of species composition from the two groups. H and O indicate home-care and OPD patients, respectively. (b) OTU heat map showing different microbiota composition between the two patient groups. Color shades indicate the relative abundance of each OTU after normalization. (c) Chao1 index showing higher species abundance in the OPD group compared to the home-care group. (d) Shannon index indicating higher species evenness in the OPD group compared to the home-care group.

**Figure 3.**
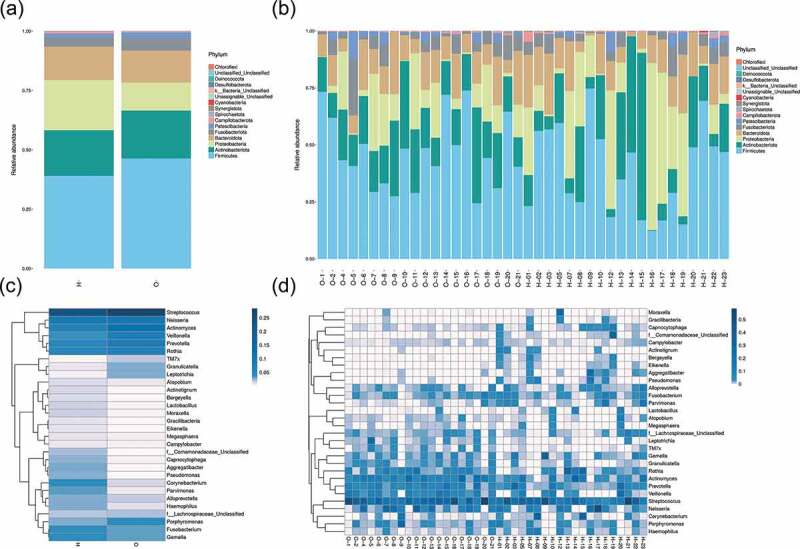
Taxonomic analysis showed distinct bacterial taxa between OPD and home-care groups. (a) Relative abundance of annotated bacterial phyla shown per group (left) and per patient (right). (b) Species distribution heat maps present similarities and differences of bacterial genera per group (left) and per patient (right). Some genera were abundant in homecare patients but not in OPD patients, and vice versa. O, OPD group; H, home-care group.

The species annotation heat map (Figure 2 and 3(b)) displayed the 30 most abundant bacterial species; the bacterial species of OPD patients were more centralized than home-care patients. Bacterial genera with similar relative abundance at the genus level in the two groups were as follows: *Streptococcus, Neisseria, Actinomyces, Veillonella, Prevotella, Rothia, Gemella, Porphyromonas*, and *Fusobacterium*. Together, the results revealed the presence of distinct bacterial taxa between the two groups.
**Figure:**
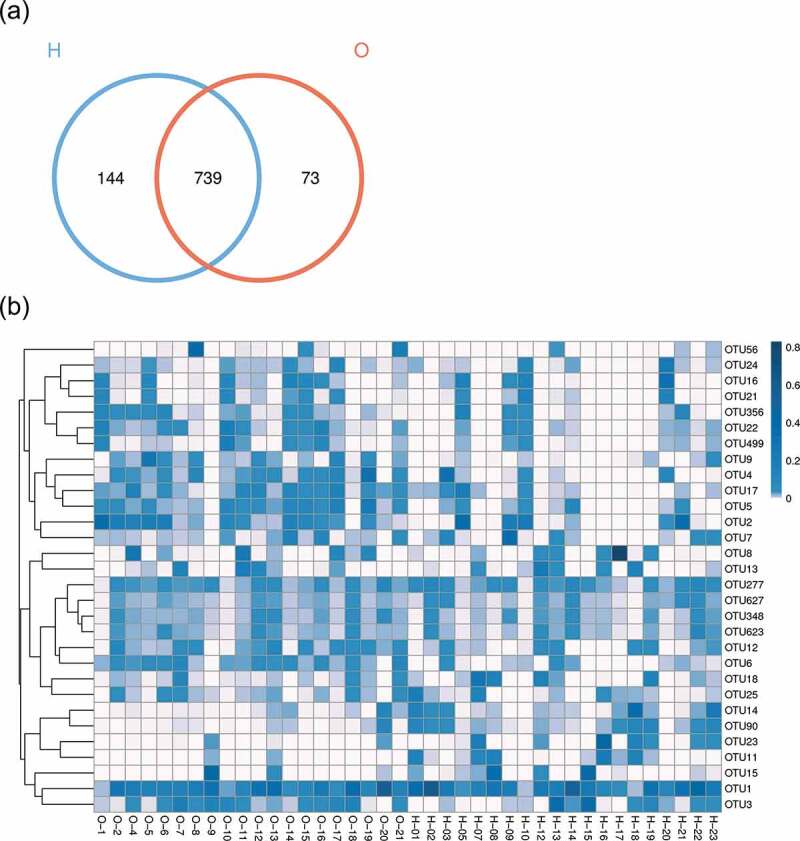

### Overall structure of bacterial communities across samples

We first made between-group comparisons and found marked differences in the oral microbiota between OPD and home-care patients. The Venn diagram (Figure 2(a)) highlights the number of OTUs that were either shared or exclusive in both groups. Specifically, 73 and 144 OTUs were exclusive in the OPD and home-care groups, respectively, while 739 OTUs were shared among both groups. The heat map (Figure 2(b)) indicated that some OTU clusters in OPD patients were not present in home-care patients and vice versa, further indicating different composition of oral microbiota between the two groups. Using Chao1 index (Figure 2(c)), the OPD patients displayed higher bacterial composition as compared to home-care patients. The Shannon index (Figure 2(d)) indicated that species evenness (i.e. homogeneity in species abundance) was higher in OPD patients than in home-care patients. The low evenness implied the existence of some dominant species in the home-care group. Overall, our data showed compositional differences in oral microbiota between OPD and home-care groups.

### Common and distinct bacterial taxa in two groups

Taxonomic analysis showed that the five most abundant phyla were *Firmicutes, Actinobacteria, Proteobacteria, Bacteroidetes*, and *Fusobacteria. Firmicutes* was the most abundant phylum, with 46.4% in OPD patients and 39.1% in home-care patients. The abundance of *Actinobacteria, Proteobacteria, Bacteroidetes*, and *Fusobacteria* was 20.2%, 11.8%, 13.5%, and 5.4%, respectively, in OPD patients, and 19.2%, 21.1%, 14.1%, and 3.9%, respectively, in home-care patients (Figure 3(a)).

### Beta diversity revealed evolutionary difference between the two groups

**Figure 4.**
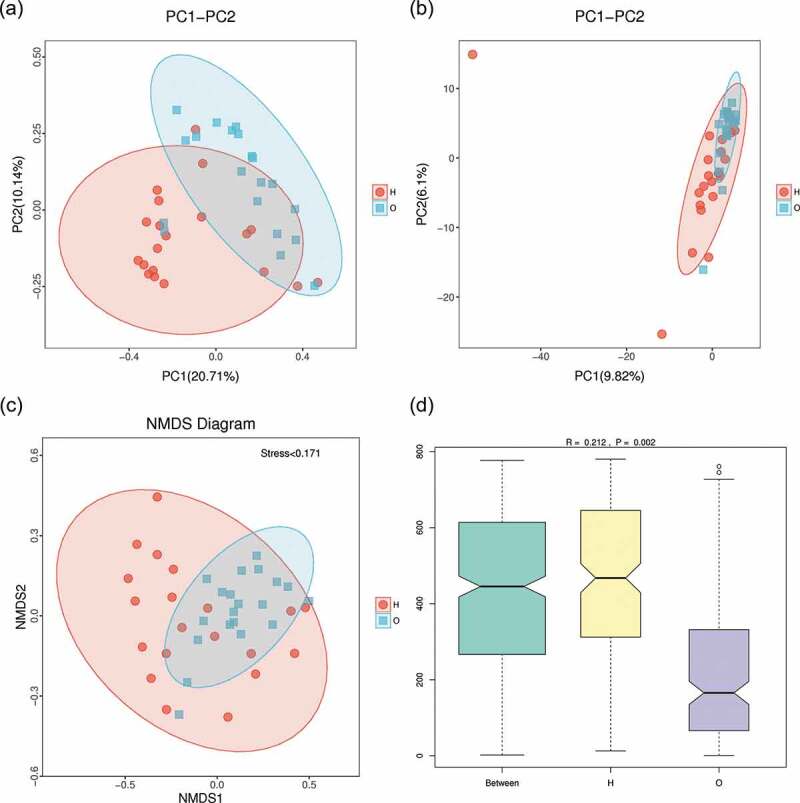
Beta-diversity and dimensionality reduction indicates a more stable microbial population among OPD patients compared to the home-care group. (a) PCoA of Bray-Curtis distance with each point representing a sample. The distance between a given pair of points indicates their similarity. (b) PCA of OTU abundances indicates variation among samples. The OPD group was more concentrated than the home-care group, indicating a more stable bacterial population. (c) NMDS plot representing differences between all samples based on Bray-Curtis dissimilarity between samples. (d) ANOSIM was used to compare ‘between group’ vs. ‘within-group’ differences. The two open circles indicate statistical significance (p-value ≤ 0.05). O, OPD group; H, home-care.

To evaluate the diversity and difference between the two groups, beta diversity (weighted unifrac analysis) was calculated. Principal coordinate analysis (PCoA) (Figure 4(a)) indicated the similarity of bacterial composition in each sample and represented the similarity between each sample. Samples of OPD patients were clustered separately from those of home-care patients, indicating their microbiota compositions were evolutionarily distinct. Principal component analysis (PCA) (Figure 4(b)) revealed that the samples from OPD patients were more concentrated and distinct from those from home-care patients, indicating a more consistent, stable bacterial population across samples from OPD patients. Concordantly, non-metric multidimensional scaling (NMDS) (Figure 4(c)) analysis revealed a significant difference in the distance of the samples from OPD patients and home-care patients. To further statistically validate the difference between the two groups, analysis of similarities (ANOSIM) (Figure 4(d)) was conducted and revealed a significant difference (R = 0.212, P = 0.002), indicating that the bacterial composition was significantly different between the two groups, and that the between-group difference was significantly greater than the within-group difference.

### Linear discriminant analysis identified distinct biomarkers between the two groups

**Figure 5.**
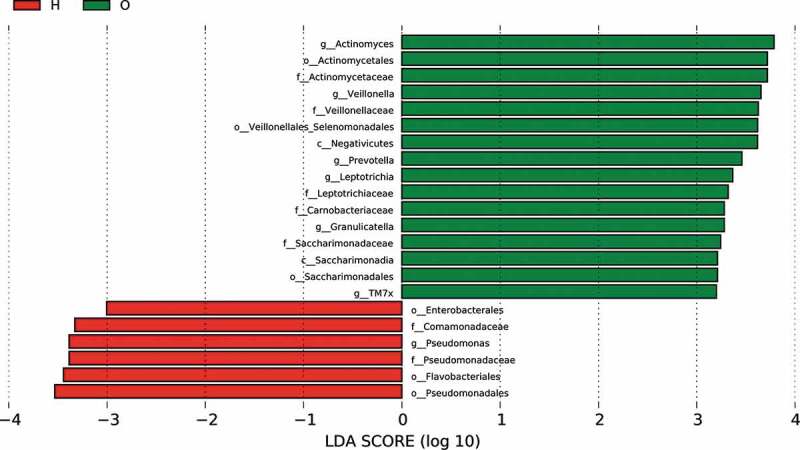
LefSe analysis showing distinct bacterial biomarkers between OPD and home-care groups. (a) Taxonomic cladogram showing distinct bacterial taxa from the two patient groups. (b) LDA scores representing significant differences in the abundance of bacterial taxa between the two groups. O, OPD group; H, home-care group.

To identify dominant bacterial species in both OPD and home-care groups, we performed linear discriminant analysis effect size (LefSe) (Figure 5(a)) and found that the dominant bacterial species in both groups were significantly different. The cladogram indicated that the most dominant bacterial species at the order level were *Actinomycetales, Veillonellales/Selenomonadales*, and *Saccharimonadales* in OPD patients, and *Enterobacteriales, Pseudomonadales*, and *Flavobacteriales* in home-care patients. Important bacterial species at the family level were *Actinomycetaceae, Veillonellaceae, Carnobacteriaceae, Leptotrichiaceae*, and *Saccharimonadaceae* in OPD patients, and *Pseudomonadaceae* and *Comamonadaceae* in home-care patients. Important bacterial species at the genus level were *Actinomyces, Prevotella, Veillonella, Granulicatella, Leptotrichia*, and TM7x in OPD patients, and *Pseudomonas* in home-care patients. According to the linear discriminant analysis (LDA) score (Figure 5(b)), in OPD patients, the orders Actinomycetales, Veillonellales/Selenomonadales, and Saccharimonadales were significantly enriched, whereas Enterobacteriales, Pseudomonadales, and Flavobacteriales were significantly depleted. At the family level, Actinomycetaceae, Veillonellaceae, Carnobacteriaceae, Leptotrichiaceae, and Saccharimonadaceae exhibited significantly higher abundance in OPD patients, whereas Pseudomonadaceae and Comamonadaceae showed a much lower abundance. At the genus level, *Actinomyces, Prevotella, Veillonella, Granulicatella, Leptotrichia*, and TM7x exhibited significantly higher abundance in OPD patients, whereas the level *Pseudomonas* was lower. Taken together, our results clearly indicated distinct dominant bacterial species between OPD and home-care patients.

**Figure:**
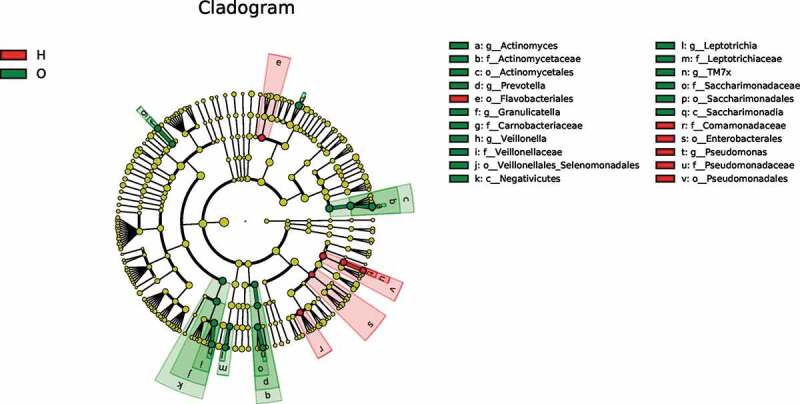

## Discussion

To our knowledge, our study was the first to use NGS to identify differences in the oral microbiota between OPD patients and home-care patients in Taiwan. Notably, both groups presented distinct bacterial diversity and homogeneity in species abundance, and their dominant bacterial species were significantly different. Within-group comparison showed that microbiota of home-care patients were more divergent than OPD patients.

The existence of some specific and dominant species may cause imbalance and dysbiosis of the oral microbiota, resulting in higher risks of oral diseases, particularly in frail populations (e.g. home-care patients). Patients who receive home care often lost their mobility and suffered from higher disabilities than OPD patients. Further, diet restriction and intake methods (e.g. oral intake or nasogastric tube) could also influence the bacterial composition of oral microbiota. In line with these, dysbiosis of the oral microbiota has been shown to be associated with multiple local and systemic human diseases, including dental caries, periodontal disease, obesity, diabetes, and cardiovascular disease [30–36]. This may also explain why patients receiving home-care have more systemic diseases and poor oral health than OPD patients.

It is possible that biomarkers identified by LefSe analysis could be used as indicators for general or oral health. For instance, the dominance of *Pseudomonas* species in the home-care patients presented a major causative factor of pneumonia. It is noteworthy that infection by more varieties of bacteria could result in more severe pneumonia as also suggested by other studies [28,37]. Our investigation correlated with previous findings in the prevalence of community-acquired pneumonia, particularly in the Asia-pacific region [37]. Intriguingly, according to patients’ history of pneumonia, our result showed that even after the recovery from pneumonia, *Pseudomonas* was not eliminated and still persisted in the oral cavity.

Factors affecting microbiota composition between different patient groups include age, physical function, general health, systemic disease, cognitive function, and indwelling devices such as urinary catheters and nasogastric tubes [38–42]. Langmore *et al*. (1998) found that factors associated with aspiration pneumonia included dependency on feeding methods, oral care, and number of decayed teeth [43]. Due to limited physical function, home-care patients suffered from severe and profound disability and were often bedridden. Our study indicated that bacterial species of bedridden patients were less abundant than those who were physically mobile. Our work was in agreement with previous studies [44–46] that higher risk of pneumonia was associated with bedridden lifestyle. Furthermore, several studies also showed that pneumonia of bedridden patients may be induced by oral care [27,47].

Given the notion that oral and general health may be affected by dysbiosis, dominant bacterial species of the two groups found in this study could serve as biomarkers to monitor and improve health quality. Taking a step further, quantitative properties (e.g. abundance or diversity) derived from oral microbiota could be used as indicators for early prevention and risk management in disease progression. We envision that dental treatments for long-term care patients should receive more comprehensive assessment of oral microbiota before initiating treatments to minimize the risk of posttreatment complications.

## Conclusion

Despite the limitation of scale in this study, we conclude that a significant difference exists in the oral microbiota between long-term care patients receiving treatment at OPD and those receiving home-care. The oral microbiota of home-care patients was less diverse than that of OPD patients, and specific pathogenic species were dominant, leading to dysbiosis.
